# Detection of *Salmonella* DNA and drug-resistance mutation by PCR-based CRISPR-lbCas12a system

**DOI:** 10.1186/s13568-023-01588-x

**Published:** 2023-09-26

**Authors:** Shan Wang, Shang Wang, Tongyu Hao, Shimao Zhu, Xinying Qiu, Yuqing Li, Xiaoxu Yang, Song Wu

**Affiliations:** 1 Institute of Urology, The Third Affiliated Hospital of Shenzhen University, Shenzhen, 518000 China; 2 The Second Affiliated Hospital of Xi’an Jiaotong University, Xi’an, China; 3 Shenzhen Institute of Synthetic Biology, Shenzhen Institutes of Advanced Technology, Chinese Academy of Sciences, Shenzhen, 518055 China; 4 South China Hospital of Shenzhen University, Shenzhen, China; 5 Medical Laboratory of Shenzhen Luohu People’s Hospital, Shenzhen, China; 6 Department of Biology and Genetics, The Medicine School of Hunan University of Chinese Medicine, Changsha, Hunan 410208 PR China

**Keywords:** *Salmonella*, Drug resistance mutation, CRISPR-lbCas12a, PCR

## Abstract

*Salmonella* is an important foodborne pathogen, which can cause serious public health problems. Rapid and accurate detection of *Salmonella* infection and drug resistance mutations in patients will provide timely guidance for clinical treatment and avoid disease progression and other related clinical problems. Here, we established a highly sensitive and quick method for *Salmonella* and drug resistance mutation detection based on polymerase chain reaction (PCR) and CRISPR-lbCas12a system and evaluated its practicability with clinical samples.

Specific CRISPR RNAs (crRNAs) and primers are designed for *Salmonella* DNA and *parC* gene S80I mutation diagnosis. CrRNAs with and without phosphorylated modification and different crRNA preparation methods are used to assess the effect on the detection system. After optimization, we detected as low as one copy of *Salmonella* DNA and drug resistance mutation *parC* S80I with the *Salmonella* DNA standard. For 94 clinical samples, this method also showed high sensitivity (100%, 95% CI: 84.98–100%) and specificity (98.48%, 95% CI: 90.73–99.92%) with less time (3 h) than plate culture (16 h) and conventional antimicrobial susceptibility testing (over 16 h). Besides, one parC S80I mutant strain was detected, which is consistent with the result of DNA sequencing. Taken together, we established a highly sensitive and specific method for *Salmonella* infection and *parC* S80I drug resistance mutation detection with fewer reagents and ordinary instruments. This assay has wide application prospects for fast detection of pathogen (bacterium and virus) infection, drug resistance determination, and proper treatment guidance.

**Supplementary Information:**

The online version contains supplementary material available at 10.1186/s13568-023-01588-x.

## Introduction

*Salmonella enterica* is an important food-borne pathogen of *Enterobacteriaceae* which may cause severe global public health problems (Zhang et al. 2018). Lots of *Salmonella* infection cases have been recorded worldwide (Kirk et al. 2015). People get infections by ingesting of contaminated foods like eggs, poultry, beef, pork, and Seafood (Zeng et al. 2019). Enteric illness in both humans and animals caused by *Salmonella* is called salmonellosis (Lutful Kabir 2010). The main symptoms of salmonellosis are vomiting, watery diarrhea, high fever, headache, abdominal pain, nausea, and loss of appetite. Children, especially young children, are more susceptible to being infected (Wang et al. 2018). If not effectively controlled, *Salmonella* infection can cause serious public health problems and economic costs. According to the World Health Organization, about 1.9 billion people worldwide suffer from diarrhea each year and 715,000 deaths. About 180 million (9%) of these diarrheal cases and 298,000 (41%) deaths were caused by *Salmonella* (WHO 2015, Besser 2018).

Conventional isolation of *Salmonella* contains nonselective pre-enrichment, plating culture with selective agars, and suspect colonies confirmation with serological or biochemical tests (J. J. Carrique-Mas and R. H. Davies 2008). Tetrathionate (TT) broth Rappaporte Vassiliadis (RV) medium has been used as official *Salmonella* enrichment media in approved standard methods. In addition, Kligler Iron Agar (KIA) is also commonly used for *Salmonella* detection in hospitals, which may take 16–24 h. In order to get results immediately, rapid *Salmonella* detection methods are needed.

Patients with normal *Salmonella* infection may recover in 3–7 days without any medical interventions. To prevent invasive salmonellosis, antimicrobial therapy is required for infants, elders, and immunocompromised patients. For more severe cases that are resistant to conventional first-line antibiotics, fluoroquinolones and third-generation cephalosporins are used. As a consequence of the widespread use of antibiotics, drug resistance emerged and became a major global food safety concern, which may increase instances of morbidity and mortality (Ma et al. 2017).

Recently, an RNA-targeting CRISPR effector Cas12a (Cpf1) exhibited an indiscriminate single-stranded DNA cleavage activity (ssDNase). Researchers have combined *Lachnospiraceae bacterium* ND2006 Cas12a (LbCas12a) with isothermal amplification for DNA detection named DETECTR (DNA endonuclease-targeted CRISPR trans reporter) which showed attomolar sensitivity (Chen et al. 2018). This study aims to establish a new nucleic acid method for *Salmonella* and drug-resistant mutation by PCR and CRISPR-lbCas12a system, which can save both time and labor.

## Materials and methods

### Nucleic acid and crRNA preparation

The 243-base pair *Salmonella pagC* gene (nucleotides 232–474, GenBank ID: 1,252,764) fragments, 260-base pair *invA* gene (nucleotides 781–1040, GenBank ID: 1,254,419) fragments, 430-base pair *parC* gene wild type and S80I mutant (nucleotides 16–446, GenBank ID: 1,254,697) sequences were synthesized by Sangon Biotech and cloned to pUC-57 vector respectively. The detailed sequences were listed in the supplementary material.

crRNAs were designed to target *Salmonella* gene *pagC*, virulence gene *invA*, and the drug resistance mutation *parC* S80I according to the protocol (Chen et al. 2018). RNA nucleotides are chemically synthesized without 5’-phosphate by Sangon Biotech, China. crRNA consists of 18nt of common sequences (repeat) for binding lbCas12a protein and 18nt for recognizing target sites (Li et al. 2018a, b), and RNA sequences are listed in Table 1.

**Table 1:** crRNA sequences used in this study

Name	Sequence (5’-3’)	Position
*invA-1*	AAUUUCUACUGUUGUAGAACCAUUUCAAUGGGAAC	869–885
*invA-2*	AAUUUCUACUGUUGUAGACUGGUUUUAGGUUUGGC	980–996
*pagC-1*	AAUUUCUACUGUUGUAGAAAUGUCGCCUUUACCGUGCC	353–372
*pagC-2*	AAUUUCUACUGUUGUAGAGUUAUACGCGCUGGCGGGUG	375–394
*parC-S80I*	AAUUUCUACUGUUGUAGAGGCGACAUCGCCUGCUAUG	246–265

Genebank ID for *pagC*, *invA*, and *parC* is 1,252,764, 1,254,419, and 1,254,697 respectively

FAM-BHQ labeled DNA probe was synthesized by Sangon Corp (China). The probe sequence and modification is 5’-FAM-TTTTTT-BHQ-3’.

### PCR

PCR was processed on an Eppendorf thermocycler with pre-denaturation at 94 °C for 5 min, followed by 35 cycles of denaturation at 94 °C for 15 s, annealing at 58 °C for 15 s, and extension at 72 °C for 15 s, 1 cycle of 72 °C for 10 min, and ending at 4 °C. The PCR reaction was performed in a 20µL system in the 0.2 mL EP tube. Each reaction contains 10µL ExTaq mix (TAKARA), 1µL forward primer (10nM) and 1µL reverse primer (10nM), 10 ng of sample DNA (or plasmid), and ddH_2_O to supplement the volume to 20µL. DNA electrophoresis was processed in 1% agarose gel in TAE buffer. Primers optimized for *pagC* and *parC* targets amplification are listed in Table S2.

### LbCas12a detection

LbCas12a detection was carried out in Buffer 3.1 (NEB), 50nM lbCas12a protein, 60nM of crRNA and 30nM labeled probe, and 5µL PCR product, and ddH_2_O to supplement the volume to 20µL. The reaction system was mixed and then incubated at 37 °C. Reactions proceeded for one hour on QuantStudio Dx (ABI) with fluorescent kinetics measured every 2 min. Each reaction was repeated in three biologically independent experiments.

### In-vitro transcription

The complete crRNA with appended T7 promoter sequences are synthesized as single-strand DNA and then annealed into double-strand DNA. The annealing process is performed in a 20µL system in the 0.2mL EP tube which contains 10µL sense template (100 nM) and 10 µL antisense template (100nM). The system was incubated at 95 °C for 5 min; then at 95ºC, for 1.5 min, and decrease 1ºC per cycle for 70 cycles, ending at 4 °C. Single-strand DNA templates are listed in Table S3. crRNAs for detecting the effect of in-vitro transcription on the lbCas12a system were obtained using HiScribe T7 Quick High Yield RNA Synthesis Kit (NEB). Each transcription system contains 10µL NTP buffer mix, 1 µg dsDNA, 2µL T7 RNA polymerase mix, and nuclease-free water to supplement the volume to 30µL. Mix thoroughly and incubate at 37 °C overnight. The transcribed crRNAs were further purified by phenol-chloroform extraction and ethanol precipitation. crRNAs were quantified by Nanodrop and stored at -80 °C before use.

### DNA extraction and quantification

*Salmonella* genomic DNA extraction from clinical stool samples is processed using the DNA extraction Kit (Tianlong Science & Technology) according to the manufacturer’s instructions. Extracted DNA samples were quantified by Nanodrop and stored at -80 °C before use.

### Direct sequencing

Nucleic acid extracts of clinical samples are PCR-amplified with *Salmonella* sense and antisense primers as listed in Table S2. Direct sequencing is conducted using the *parC* antisense primer (*parC*-1r) by Guangzhou IGE Biotechnology LTD.

### Sample information

Stool samples are obtained from each donor and stored at − 80 °C until processing. To confirm whether the PCR-lbCas12a assay can be applied for clinical detection, nucleic acid extracts derived from 94 stool samples from the pediatric of Shenzhen Luohu People’s Hospital (Sept. 2021 to April 2022). The basic information of these patients is available (age < 9; mean age, 2.7 ± 2.3 years; 39 girls and 55 boys, Table S4).

### Statistical analysis

Means and standard deviations are calculated by GraphPad Prism software version 5 (GraphPad, Inc., La Jolla, CA, USA). Mean differences in quantification are determined by paired *t*-test. All statistical tests are two-sided, and *P* < 0.05 is considered statistically significant.

### Study approval

The Medical Ethics Committee of the Shenzhen Luohu hospital approved the study (2021-LHQRMYY-KYLL-039).

## Results

### Establishment of PCR-lbCas12 DETECTR

**Fig. 1:**
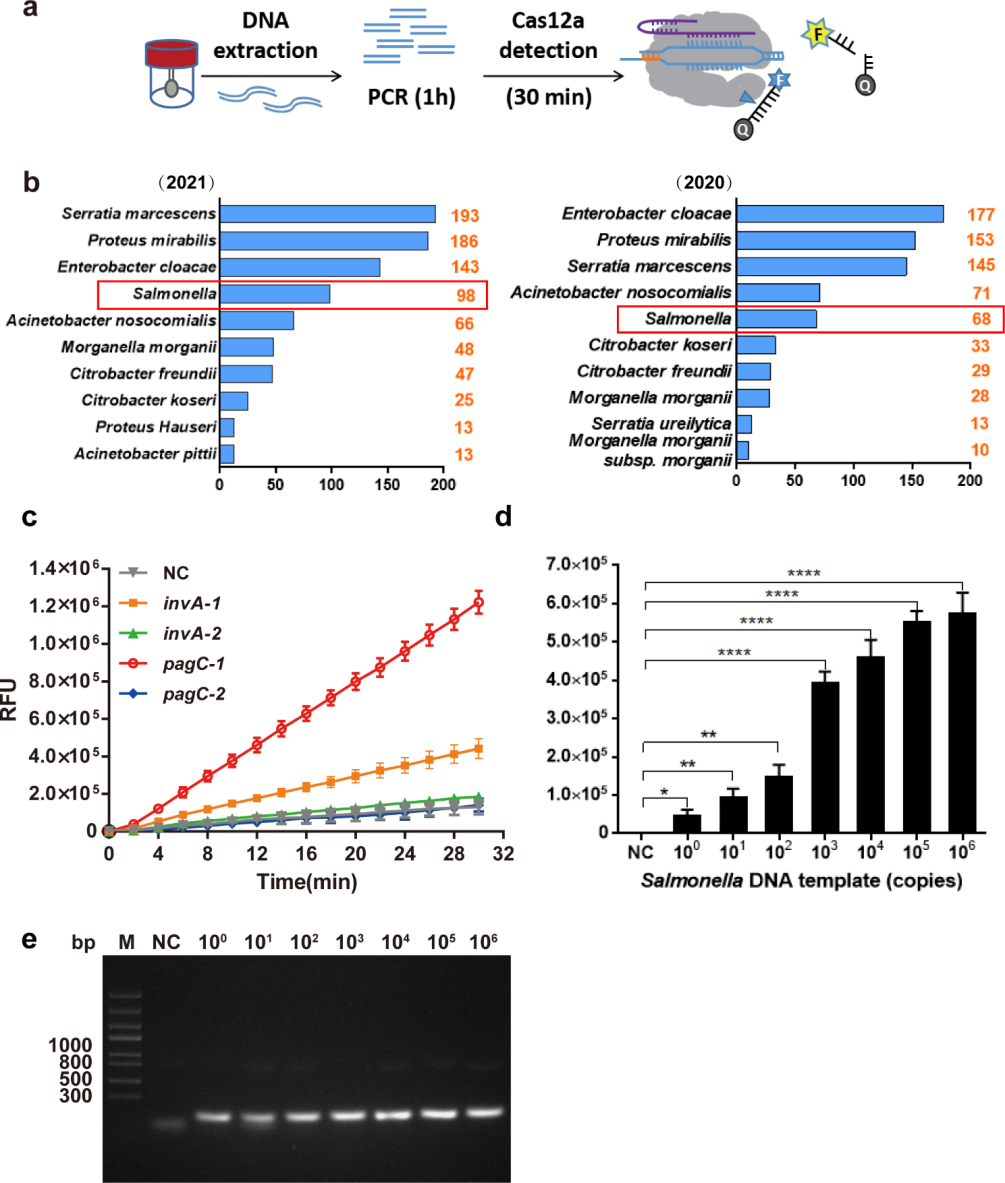
Establishment of PCR-lbCas12a assay for *Salmonella* DNA detection. (**a**) Schematic of PCR-lbCas12a. (**b**) *Positive ranking of G*^*−*^*bacilli of* Shenzhen Luohu Hospital Group *in 2021(left) and 2020(right).* (**c**) Screening of crRNAs for *Salmonella* DNA detection by lbCas12a collateral detection. Represent one of three independent experiments. (**d**) PCR-lbCas12a can detect *Salmonella* at concentrations of 1 copy in 30 min through the lbCas12a cleavage reaction (n = 3 technical replicates; *, *p* < 0.05, **, *p* < 0.01, ****, *p* < 0.0001; bars represent mean ± s.e.m.). (**e**) Agarose gel electrophoresis for the PCR products of different *Salmonella* standard DNA (copies)

The whole detection process is illustrated in Fig. 1a. According to the data of infected persons from the Medical Laboratory of Shenzhen Luohu People’s Hospital, *Salmonella is always among the top 5 positive Gram-negative (G*^*−*^*) bacilli in both 2021 and 2020* (Fig. 1b). To obtain efficient and specific crRNAs applicable for detecting *Salmonella* DNA, we used blast to screen the most suitable genes and target sequences. Results showed that *invA* and *pagC* are both specific and conservative genome fragments. We selected 4 candidate crRNAs (*invA*-1,2 and *pagC*-1,2 sequences were listed in Table 1) in the conserved region of the two genes. Single-stranded DNA sequences of about 38-40nt were synthesized as templates (Table S1) to test the fluorescence signal. Among the 4 crRNA sequences, *pagC*-1 showed the most powerful signal (Fig. 1c), which will be selected as the following detection target. To select the best primer for *pagC*-1 target amplification, 4 pairs of PCR primers were further designed (Table S2) and primers *pagC* 3f, 3r showed the strongest amplification efficiency (Fig. S1a).

Using serial dilutions of the *Salmonella* DNA standard, we found that as few as one copy per test of *Salmonella* DNA could be detected with *pagC*-1 crRNA in 30 min after the PCR amplification step (Fig. 1d). This is more sensitive than that of the agarose gel electrophoresis assay, for the DNA bands of low templates (10^0^-10^1^ copies) were sometimes not clear enough to identify (Fig. 1e).

### Effects of phosphorylation and in vitro transcription of crRNA on Cas12a system

**Fig. 2:**
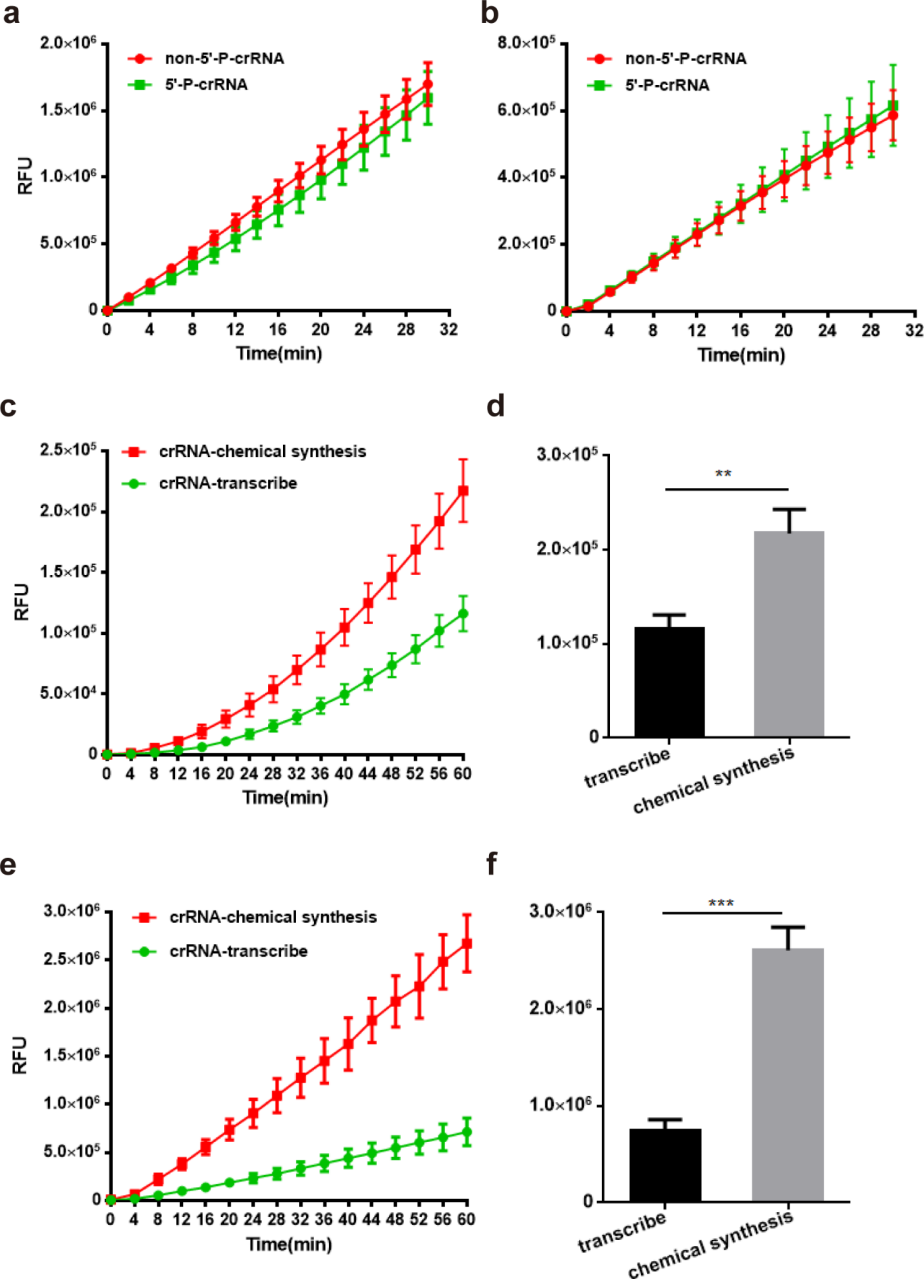
Detection efficacy of crRNAs with different preparation methods and modification. (**a**), (**b**) Comparation of detection curve between crRNAs (*pagC*1: left and *invA*1: right) with and without 5’ -phosphate. (**c**) Comparation of detection curve between *pagC*2-crRNAs obtained by chemical synthesis and in-vitro transcription. (n = 3 technical replicates; bars represent mean ± s.e.m.). (**d**) Fluorescence signal of chemically synthesized pagC2-crRNA is significantly higher than that in-vitro transcribed at 60 min (n = 3 technical replicates; **, *p* < 0.01, bars represent mean ± s.e.m.). (**e**) Comparation of detection curve between *invA*1-crRNAs obtained by chemical synthesis and in-vitro transcription. (n = 3 technical replicates; bars represent mean ± s.e.m.), (**f**) Fluorescence signal of chemically synthesized invA1-crRNA is significantly higher than that in-vitro transcribed at 60 min (n = 3 technical replicates; ***, *p* < 0.001, bars represent mean ± s.e.m.)

CRISPR-Cas12a system used 32-42nt crRNA which could be easily obtained by chemosynthesis and in-vitro transcription with T7 RNA polymerase. Naturally transcribed RNA has a 5’-phosphate group, whereas the chemically synthesized RNA usually lacks this modification. Crystal structure study shows that certain proteins (Siwi and prokaryotic AGO-clade Argonautes) recognize piRNA in a 5’-phosphate-dependent manner, which indicates the importance of RNA 5’-phosphate (Matsumoto et al. 2016). However, there is no study on the effect of crRNA modification on the lbCas12a system. So we compared the fluorescence signals for crRNAs with and without 5’-phosphorylation. For the same crRNA sequences, the lbCas12a system showed no significant difference for both crRNAs with and without 5’-phosphorylation (Fig. 2a, b).

With the development of molecular biology techniques, RNA sequences up to 100nt can be chemically synthesized. In this study, we investigated the effect of different crRNA preparation methods such as chemical synthesis (solid phase synthesis with phosphotriester technique) and in-vitro transcription on the lbCas12a system. RNAs that are chemically synthesized require about 30 h: 10 h for synthesis of crude RNA product, 10–15 h for cutting chemical protective base, and 1–3 h for HPLC (High Performance Liquid Chromatography) purification. The purity of the finally obtained RNA is over 90%. The process for in-vitro transcription is about 15 h: 3 h for DNA templates annealing, 6–10 h for transcription, and 2 h for phenol-chloroform extraction and ethanol precipitation (Table S3 shows the transcription template sequences). Obviously, in-vitro transcription costs less time than chemosynthesis. However, as is shown in Fig. 2, the fluorescence curve of chemically synthesized crRNAs exhibited a relatively stronger signal compared to that transcribed in vitro (Fig. 2c, d shows crRNA-*pagC*2, Fig. 2e, f shows crRNA-*invA*2). As time passes, the difference in signal becomes more pronounced. At 60 min, both chemosynthesis crRNAs (*pagC2* and *invA1*) are significantly higher than those transcribed in vitro.

These results indicated that 5’-phosphorylation of crRNA sequences had no obvious effect on their signal. With the same sequence and concentration, in-vitro transcribed crRNAs displayed a weaker signal, which may be attributed to the lack of strict quality control for the crRNA: the length and concentration of crRNAs produced by in-vitro transcription are not stable enough, and the purity of chemo-synthesized crRNAs is relatively higher.

### Drug-resistant mutations can be sensitively detected by PCR-lbCas12a DETECTR

**Fig. 3:**
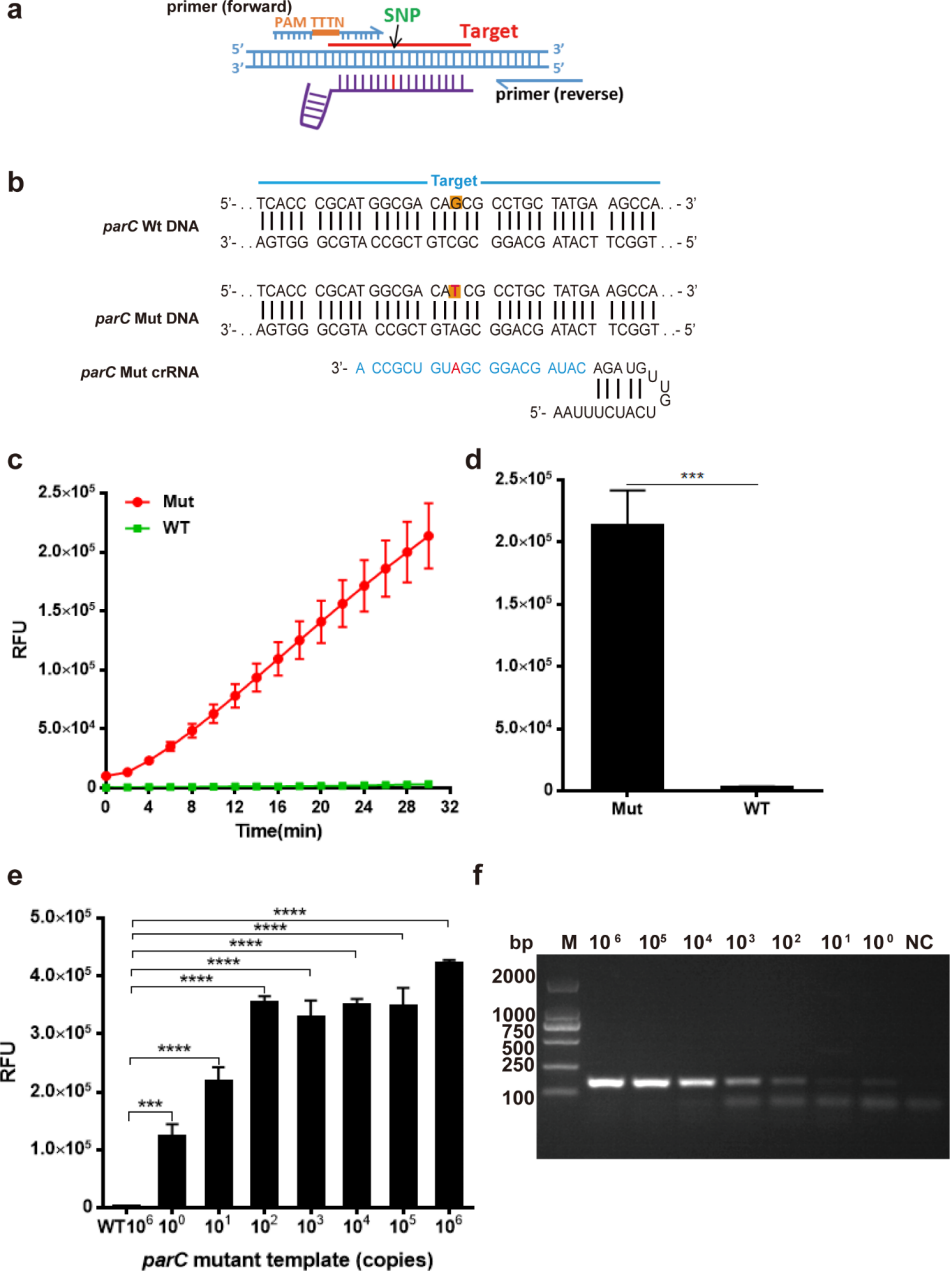
PCR-lbCas12a can be used to identify the drug resistant mutation. (**a**) Schematic of PCR primer design for the mutant site lack of PAM sequence. (**b**) Schematic of mutant regions and crRNA sequences used for detection. Mutant site in the target are highlighted as orange; crRNA for mutant sequence detection is colored as red. (**c**, **d**) Specificity detection of *parC* S80I crRNA. (n = 3 technical replicates; ***, *p* < 0.001; bars represent mean ± s.e.m.) (**e**) PCR-lbCas12a can detect *Salmonella parC* S80I at concentrations of 1 copy in 30 min (n = 3 technical replicates; ****, *p* < 0.0001; bars represent mean ± s.e.m.). (**f**) The electropherogram of the PCR product with gradient dilution plasmid template (copies)

Ciprofloxacin-resistant *Salmonella* was found to be widely distributed in various animal-derived food products (Sharma et al. 2019). The most commonly detected mutations were T57S and S80I in *parC*, as well as gyrA mutations S83F and D87N/Y (Chen et al. 2021). Here, we used PCR-lbCas12a to detect the drug resistance-related mutation *parC* S80I in the *Salmonella* genome. After the sequence analysis, no PAM (TTTN) was found near the mutant site. Primers for PCR amplification were intentionally designed to introduce a nearby PAM sequence nearby the mutation, allowing for sequence-independent detection of any mutant site. So 8nt that base-pair the target fused with TTTN was used as forward primers to amplify the DNA fragment and tag the DNA fragment with PAM sequence (Fig. 3a and Table S2). After the primer screening, the *parC* S80I target was successfully PCR amplified with a single and clear electrophoresis band (Fig. S1b).

We further designed the crRNA that exactly matches the S80I mutant sequence with only one base difference from the wild-type sequence (Fig. 3b). To confirm the crRNA’s activity, we used ssDNA of wild-type and mutant sequences fused with CAAA (reverse complementary with TTTG) as templates, respectively (Table S1). The specificity of this crRNA was initially detected. According to the fluorescence curve, *parC* S80I crRNA exhibited a specific fluorenscence signal to the mutant sequence, while the wild-type template showed no obvious increase (Fig. 3c). Based on the 30-minute data, the fluorenscence signal of the mutant sequence is significantly higher than that of the wild type. (Fig. 3d). To determine the lower limit of PCR-lbCas12a for detecting drug-resistant mutations, we used diluted S80I standard plasmids as templates and performed detection with *parC* S80I crRNA. Based on its much higher FAM signal after a 30-minute reaction, we were able to distinguish the *Salmonella* DNA with the S80I mutation (1 copy) from the wild-type plasmid (10^6^ copies) per test (Fig. 3e). Additionally, we assessed the primer’s efficacy through agarose gel electrophoresis. (Fig. 3f). Similar to *pagC* target amplification, the electrophoresis band is blurry to identify especially for templates with low DNA copies (10^0^-10^3^ copies).

To summarize, our method is sensitive, reliable, and highly distinguishable, enabling accurate identification of drug-resistant sequences with only single-base mutation from wild-type sequences.

### Clinical sample detection for *Salmonella* DNA and drug-resistant mutations

**Fig. 4:**
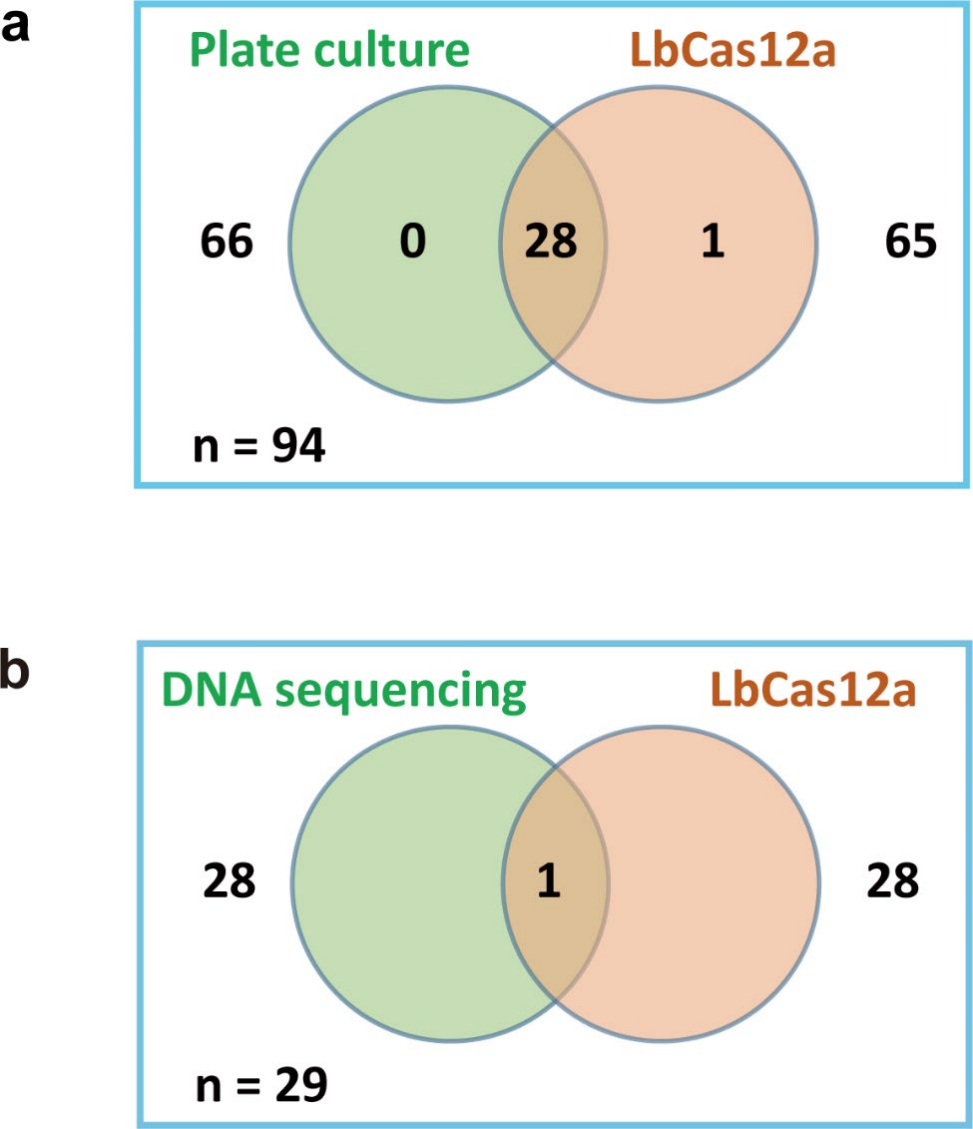
PCR-lbCas12a can successfully detect the *Salmonella* DNA and *parC* S80I mutation in clinical samples. (**a**) Venn diagrams comparing *Salmonella* DNA detection results of PCR-lbCas12a and KIA plate culture for 94 clinical stool samples; (**b**) Venn diagrams comparing *Salmonella parC* S80I detection results of PCR-lbCas12a and direct sequencing for stool nucleic acid extracts of 29 *Salmonella* positive samples

To apply this technology in clinical practice, we collected 94 children’s stool samples that had been diagnosed and confirmed by KIA (Kligler’s Iron Agar test) examination conducted by the laboratory department. Clinical sample information and detection results were listed in Table S4. Using the lbCas12a system, we successfully detected *Salmonella* from 28 positive samples which was consistent with plate culture (sensitivity: 100%, 95% CI: 84.98–100%) (Fig. 4a). Furthermore, we detected one positive sample for *Salmonella* out of 66 negative samples (specificity: 98.48%, 95% CI: 90.73–99.92%). To further validate this case, we subjected the nested PCR products to Sanger sequencing using *Salmonella*-specific primers. *T*he sequencing showed a positive signal and the blast results totally match the *Salmonella genome*. This means the sample was indeed positive while being falsely diagnosed as a negative case. Similar results were also observed by the RPA system combined with lbCas12a named DETECTR.

As previously described, *parC* gene S80I is one of the most common mutations that cause ciprofloxacin resistance. Direct sequencing and PCR-lbCas12a are applied to 29 positive samples confirmed by previous detection. As shown in Fig. 4b, one sample with *parC* S80I mutation was detected by both direct sequencing and PCR-lbCas12a. These results indicated that PCR-lbCas12a can also accurately detect the mutation with a single base difference in clinical samples like direct sequencing.

## Discussion

With the development of nucleic acid testing (NAT) technology, rapid detection is getting more and more attention. Especially for infectious pathogens, fast and accurate virus detection is urgently needed. The CRISPR-Cas12a system used in DETECTR or HOLMES (an one-HOur Low-cost Multipurpose highly Efficient System) showed great potential for DNA detection (Chen et al. 2018; Li et al. 2018a, b). Using three signal amplification steps, Qiao et al. developed a BCA-RPA-Cas12a system for *Salmonella typhimurium* detection, which can detect as low as single-digit *S. typhimurium* cells in milk samples (Cai et al. 2021). In our study, we combined PCR amplification and the advantages of lbCas12a for *Salmonella* and drug-resistance mutation detection. This method also shows high sensitivity and specificity but requires fewer reagents, which is convenient for clinical detection and application. Besides that, it only needs ordinary instruments (fluorescence ration PCR instrument) and costs less time (about 3 h) than conventional plate culture (16–24 h), which is especially needed for infants, young children, and immunocompromised people with severe symptoms in order to get treatment guidance timely.

5’-Terminal modifications play pivotal roles in the maturation, function, and turnover of cellular RNAs. For bacteria, the triphosphate initially present at the 5’ end can protect its transcripts from degradation (D. J. Luciano and J. G. Belasco 2019). 5’-phosphate of single-stranded DNA or RNA is important for RNP complex loading (Swarts et al. 2014; Matsumoto et al. 2016), but the essence of crRNA 5’-phosphorylation in lbCas12a system has not been studied. In this study, we found that crRNAs with 5’-phosphate had no obvious impact on the lbCas12a detection assay. We supposed that 5’-phosphate didn’t affect crRNAs’ binding activity to lbCas12a protein and further target recognition. However, crRNAs obtained from different preparation methods varied a little: signals of in-vitro transcribed crRNAs are weaker than that chemically synthesized. We speculated that the quality and purity of the crRNA in-vitro transcribed were relatively lower than chemosynthesis and HPLC purification. Besides, several factors can lead to premature termination of transcripts during in-vitro transcription, such as the presence of terminator-like sequences in the template or other sequences that are difficult to transcribe (B. Beckert and B. Masquida 2011). So we speculated that in-vitro transcription may obtain a mixture of crRNAs with different lengths (only partly full-length crRNAs), which resulted in different signals. In order to guarantee the stability and accuracy of the assay, chemically synthesized crRNAs are recommended.

For clinical sample detection, the lb*Cas12a* system shows higher sensitivity than conventional plate culture: all samples (28/94) that tested Salmonella positive by plate culture were also identified by PCR-lbCas12a; one *Salmonella* positive clinical sample with a negative report from plate culture was detected by our assay and further confirmed by direct sequencing of the nest PCR product with *invA* primer. We supposed it may be due to the low bacterial concentration or weak bacterial vitality that led to an indistinctly chromogenic reaction. However, PCR-lbCas12a can detect as low as one copy of *Salmonella* DNA which is not affected by the vitality of pathogenic bacteria. It follows from the above that PCR-lbCas12a exhibits great potential for *Salmonella* DNA in clinical samples.

For mutation sites with TTTN ahead, crRNA is exactly after the PAM sequence. When there is no TTTN sequence in front of the mutant base, an appropriate primer design can introduce the PAM sequence in the PCR products. In our study, we successfully designed PCR primers for S80I mutation amplification and PAM sequence introduction, which greatly improved the applicability of the lbCas12a system as the former study described (Li et al. 2018a, b). But, due to the limited *Salmonella-positive samples, we only detected one parC S80I* drug resistance strain. To further confirm the reliability, a large number of positive clinical samples are still needed. Using appropriate antibiotics in pre-enrichment and selective media, antimicrobial-resistant Salmonella could be recovered and detected (J. J. Carrique-Mas and R. H. Davies 2008). Conventional antimicrobial susceptibilities testing like broth macrodilution and microdilution tests, the disk diffusion tests, also requires at least 16 h of incubation (J. H. Jorgensen and M. J. Ferraro 1998, Ren et al. 2020). Our study provided a fast method for *Salmonella* DNA and drug resistance mutation detection which may give aid to rational drug use while minimizing the toxicity of drugs that may no longer be effective. Although our study draws on existing methods (*DETECTR or HOLMES)* and the lbCas12a system, suitable crRNAs for specific pathogens need sequence alignment and analysis, further selection, and optimization. This method can further be applied not only for fast diagnosis of a variety of pathogenic bacteria, viruses, and fungi in clinical samples but also for the detection of pathogenic microorganisms in food and environmental samples.

### Electronic supplementary material

Below is the link to the electronic supplementary material.

Supplementary Material 1

## Data Availability

All data generated or analyzed during this study are included in this published article and supplementary file.

## List of abbreviations

crRNA: Specific CRISPR RNA
KIA: Kligler Iron Agar
DETECTR: DNA endonuclease-targeted CRISPR trans reporter
HOLMES: an one-HOur Low-cost Multipurpose highly Efficient System
