# Supplementary material for: Detection of Salmonella DNA and drug-resistance mutation by PCR-based CRISPR-lbCas12a system
Source: AMB Express. 2023 Sep 26;13:100. doi: 10.1186/s13568-023-01588-x (PMC10522547; doi:10.1186/s13568-023-01588-x)
Supplement: Supplementary file 1 — Supplementary Material 1 [file 13568_2023_1588_MOESM1_ESM.docx]

**Supplementary data for**

**Detection of *Salmonella* DNA and drug-resistance mutation by PCR-based CRISPR-lbCas12a system**

Shan Wang*^1,2^, Shang Wang*^1,3^ , Tongyu Hao*^5^, Shimao Zhu^1,4^, Xinying Qiu^5^, Yuqing Li^1,4^, Xiaoxu Yang^6^, Song Wu^#1,4^

^1^Institute of Urology, The Third Affiliated Hospital of Shenzhen University, Shenzhen 518000, China.

^2^ The Second Affiliated Hospital of Xi'an Jiaotong University, Xi'an, China.

^3^Shenzhen Institute of Synthetic Biology, Shenzhen Institutes of Advanced Technology, Chinese Academy of Sciences, Shenzhen 518055, China.

^4^ South China Hospital of Shenzhen University, Shenzhen, China.

^5^ Medical Laboratory of Shenzhen Luohu People’s Hospital, Shenzhen, China.

^6^Department of Biology and Genetics, The Medicine School of Hunan University of Chinese Medicine, Changsha, Hunan, 410208, PR China.

Corresponding author: Song Wu, Ph.D., [wusong@mail.szu.edu.cn](mailto:wusong@mail.szu.edu.cn;Yuanzhong);

#The authors wish it to be known that, in their opinion, the first 3 authors should be regarded as joint First Authors.

**Supplementary materials and methods**

*Nucleic Acid sequence*

Standard DNA carrying *Salmonella* *pagC* and *invA* gene fragments were synthesized and cloned to pUC-57 vector. Full sequences are as follows:

5’-GTCTGTTGAGCCTGAAGGTATTCATTACCATGACAAGTTTGAGGTGAAGTACGGTTCTTTAATGGTTGGGCCAGCCTATCGATTGTCTGACAATTTTTCGTTATACGCGCTGGCGGGTGTCGGCACGGTAAAGGCGACATTTAAAGAACATTCCACTCAGGATGGCGATTCTTTTTCTAACAAAATTTCCTCAAGGAAAACGGGATTTGCCTGGGGCGCGGGTGTACAGATGAATCCGCTGGA (*pagC*)

CGTAAATGGCGATAGCGATAATATGGGGCGGAATATCATGACGCAGCTGTTGAACAACCCATTTGTATTGGTTGTTACGGCTATTTTGACCATTTCAATGGGAACTCTGCCGGGATTCCCACTGCCGGTTTTTGTTATTTTATCGGTGGTTTTAAGCGTACTCTTCTATTTTAAATTCCGTGAAGCAAAACGTAGCGCCGCCAAACCTAAAACCAGCAAAGGCGAGCAGCCGCTCAGTATTGAGGAAAAAGAAGGGTCGT (*invA*) -3’

For drug resistance detection, wild type and mutant *Salmonella parC* gene fragments were synthesized and cloned to pUC-57 vector respectively. Sequences are as follows with mutant sites labeled in brackets:

5’-ATGAGCGATATGGCAGAGCGCCTTGCGCTACATGAATTTACGGAAAACGCCTACTTAAACTACTCCATGTACGTGATCATGGATCGTGCGTTGCCGTTTATTGGCGACGGCCTGAAGCCGGTACAGCGCCGCATCGTCTATGCGATGTCAGAGCTGGGGCTGAACGCCACCGCTAAATTTAAAAAATCCGCCCGTACCGTTGGTGACGTACTGGGTAAGTATCACCCGCATGGCGACAGC（**S80I：AGC→AtC**）GCCTGCTATGAAGCCATGGTGCTGATGGCGCAGCCGTTCTCTTACCGTTACCCGCTGGTCGATGGCCAGGGGAACTGGGGCGCGCCGGATGATCCGAAGTCATTCGCGGCGATGCGTTATACCGAATCCCGCCTGTCCAAATACGCCGAGCTGCTGTTAAGCGAACTCGGTCAGGGGACGGCGGACTGGG (*parC*)-3’

Genebank ID：1254697.

**Supplementary Text**

For different targets’ PCR amplification, we designed more than one pair of primers. After agarose gel [electrophoresis](http://www.baidu.com/link?url=MwKEV8EqKLUZnW7fh0mYxUwPb9BCfJD9Z3iMPGVM7KEBgSLR6I_Is_kf4hp2MrDPAsd6ubDGjWocA1E0_B3Shnh1CnjEfzvBAv1mUP9oBLw7dUeO4-3NJQRnWF9_DQTH) of the PCR products, we selected primers with the highest amplification efficiency for subsequent experiments. (**Fig. S1**).

**FIGURE LEGENDS**

**
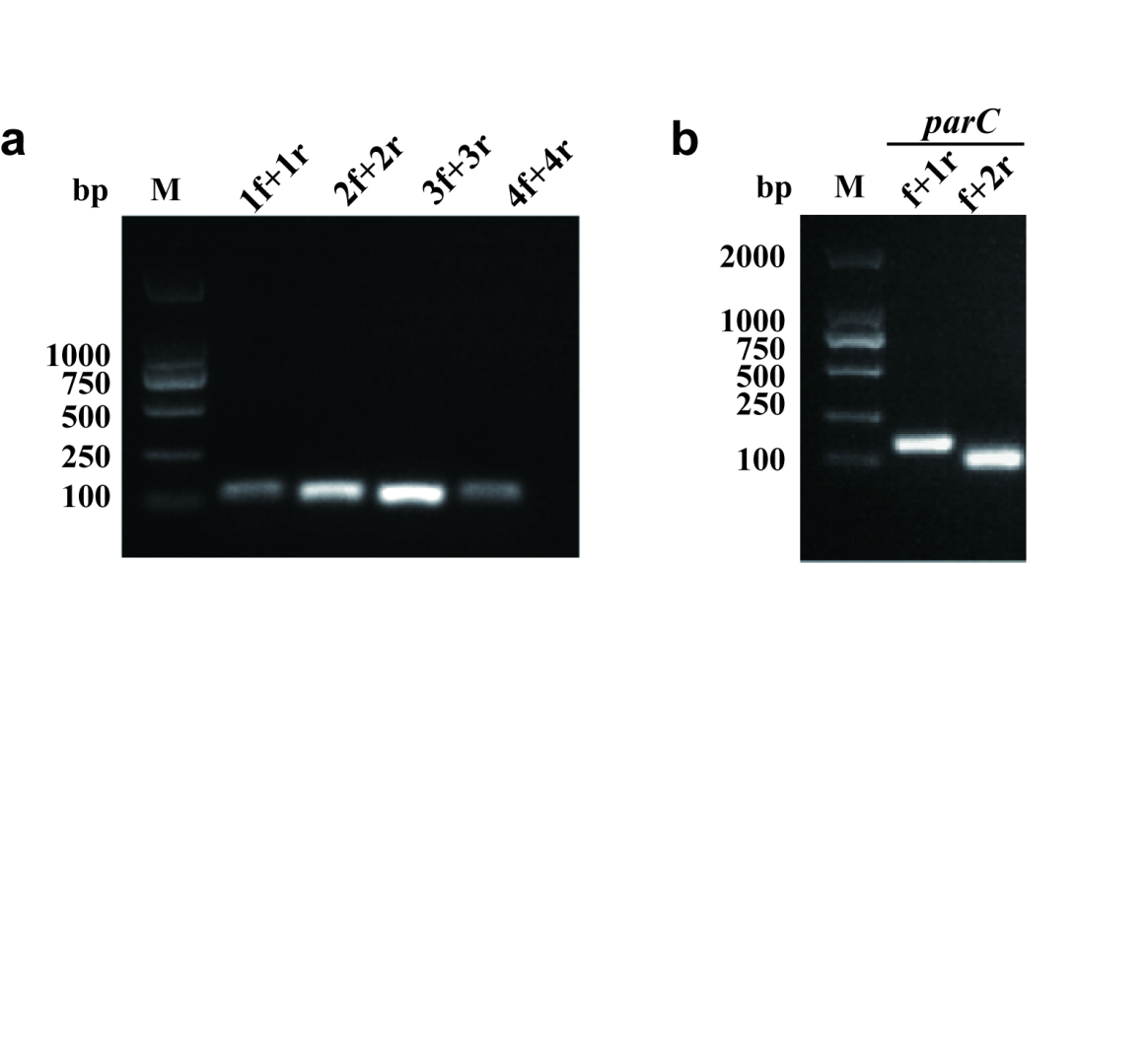
**

**Fig. S1** Primer screen for *pagC* and *parC* gene amplification

1. Comparison of primer amplification efficiency for *pagC* target;
2. Comparison of primer amplification efficiency for *parC* target.

**Supplementary Tables.**

**Table S1. ssDNAs used in this study.**

| Name | Sequence （5’-3’） |
| --- | --- |
| *invA*-1-ssDNA | TCCCGGCAGAGTTCCCATTGAAATGGTCAAAATAGCC |
| *invA*-2-ssDNA | ACGTAGCGCCGCCAAACCTAAAACCAGCAAAGGCGAG |
| *pagC*-1-ssDNA | GGCGGGTGTCGGCACGGTAAAGGCGACATTTAAAGAACAT |
| *pagC*-2-ssDNA | ACCGTGCCGACACCCGCCAGCGCGTATAACGAAAAATTGT |
| *parC*-Wt-ssDNA | ACCATGGCTTCATAGCAGGCGCTGTCGCCACAAAGGTGAT |
| *parC*-Mut-ssDNA | ACCATGGCTTCATAGCAGGCG**A**TGTCGCCACAAAGGTGAT |

**Table S2. Primers used in this study.**

| Primer | Sequence （5’-3’） | Position | Product size ( bp ) |
| --- | --- | --- | --- |
| *pagC*-1f | TTTTCGTTATACGCGCTGGC | 326-345 | 121 |
| *pagC*-1r | CCCAGGCAAATCCCGTTTTC | 427-446 |  |
| *pagC*-2f | ACGGTTCTTTAATGGTTGGGC | 282-302 | 117 |
| *pagC*-2r | CGCCATCCTGAGTGGAATGT | 379-398 |  |
| *pagC*-3f | GGTTCTTTAATGGTTGGGCCAG | 284-305 | 119 |
| *pagC* -3r | GAATCGCCATCCTGAGTGGA | 383-402 |  |
| *pagC* -4f | TTTTTCGTTATACGCGCTGGC | 325-345 | 123 |
| *pagC* -4r | CCCCAGGCAAATCCCGTTT | 429-447 |  |
| *parC*-f | GGGTAAGTATCACC**TTTG**TGGCGAC | 213-237 |  |
| *parC*-1r | GCATCGCCGCGAATGACTTC | 336-355 | 142 |
| *parC*-2r | GACCAGCGGGTAACGGTAAGAG | 279-300 | 87 |

**Table S3. Transcriptional templates used in this study.**

| Name | Sequence （5’-3’） |
| --- | --- |
| trans-*pagC2*-f | TAATACGACTCACTATAGGG  AATTTCTACTGTTGTAGAGTTATACGCGCTGGCGGGTG |
| trans-*pagC2*-r | CACCCGCCAGCGCGTATAACTCTACAACAGTAGAAATT  CCCTATAGTGAGTCGTATTA |
| trans-*invA*1-f | TAATACGACTCACTATAGGG  AATTTCTACTGTTGTAGAACCATTTCAATGGGAAC |
| trans-*invA*1-r | GTTCCCATTGAAATGGTACATTTCTACAACAGTAGAAATT  CCCTATAGTGAGTCGTATTA |

**Table S4. Clinical sample information.**

| **Sample No.** | **Genders** | **Age** | **Date of  Sapling** | **Plate  culture** | **CRISPR- Cas12a** | **Sample No.** | **Genders** | **Age** | **Date of  Sapling** | **Plate  culture** | **CRISPR- Cas12a** | **Sample No.** | **Genders** | **Age** | **Date of  Sapling** | **Plate  culture** | **CRISPR- Cas12a** |
| --- | --- | --- | --- | --- | --- | --- | --- | --- | --- | --- | --- | --- | --- | --- | --- | --- | --- |
| **S1** | **M** | **0.5** | **2021/11/23** | **+** | **+** | **S33** | **F** | **2** | **2021/9/26** | **-** | **-** | **S64** | **F** | **4** | **2021/12/18** | - | - |
| **S2** | **F** | **0.5** | **2021/10/4** | **-** | **-** | **S34** | **M** | **1** | **2021/10/2** | **-** | **-** | **S65** | **M** | **4** | **2021/12/12** | - | - |
| **S3** | **M** | **0.5** | **2021/11/21** | **+** | **+** | **S35** | **F** | **1** | **2021/10/3** | **+** | **+** | **S66** | **F** | **3** | **2021/12/13** | **-** | **-** |
| **S4** | **F** | **2** | **2021/10/4** | **+** | **+** | **S36** | **F** | **3** | **2021/10/1** | **-** | **-** | **S67** | **F** | **3** | **2021/12/16** | - | - |
| **S5** | **F** | **8** | **2021/9/22** | **+** | **+** | **S37** | **M** | **1** | **2021/10/5** | **-** | **-** | **S68** | **M** | **1** | **2021/12/20** | - | - |
| **S6** | **M** | **1** | **2021/9/27** | **-** | **+** | **S38** | **M** | **2** | **2021/9/27** | **-** | **-** | **S69** | **M** | **1** | **2021/12/21** | - | - |
| **S7** | **M** | **0.5** | **2021/11/20** | **-** | **-** | **S39** | **M** | **1** | **2021/9/26** | **-** | **-** | **S70** | **M** | **2** | **2022/1/8** | + | + |
| **S8** | **M** | **0.5** | **2021/11/19** | **-** | **-** | **S40** | **M** | **4** | **2021/10/5** | **-** | **-** | **S71** | **M** | **1** | **2022/1/8** | - | - |
| **S9** | **F** | **0.5** | **2021/11/17** | **-** | **-** | **S41** | **M** | **8** | **2021/10/5** | **-** | **-** | **S72** | **F** | **1** | **2022/1/10** | - | - |
| **S10** | **F** | **1** | **2021/10/1** | **+** | **+** | **S42** | **M** | **1** | **2021/10/4** | **-** | **-** | **S73** | **F** | **3** | **2022/1/12** | - | - |
| **S11** | **M** | **3** | **2021/9/27** | **+** | **+** | **S43** | **F** | **0.5** | **2021/11/26** | **+** | **+** | **S74** | **M** | **2** | **2022/1/14** | **-** | **-** |
| **S12** | **M** | **6** | **2021/10/1** | **+** | **+** | **S44** | **M** | **1.5** | **2021/11/26** | **+** | **+** | **S75** | **F** | **1** | **2021/12/24** | - | - |
| **S13** | **F** | **1** | **2021/9/17** | **+** | **+** | **S45** | **M** | **2** | **2021/12/2** | **+** | **+** | **S76** | **F** | **8** | **2022/12/25** | - | - |
| **S14** | **F** | **9** | **2021/9/27** | **+** | **+** | **S46** | **F** | **4** | **2021/12/3** | **+** | **+** | **S77** | **M** | **9** | **2022/12/28** | - | - |
| **S15** | **M** | **0.6** | **2021/9/12** | **+** | **+** | **S47** | **F** | **1** | **2021/12/6** | **+** | **+** | **S78** | **F** | **4** | **2022/1/10** | **-** | **-** |
| **S16** | **M** | **2** | **2021/9/24** | **+** | **+** | **S48** | **M** | **6** | **2021/12/2** | **+** | **+** | **S79** | **M** | **6** | **2022/1/12** | - | - |
| **S17** | **M** | **4** | **2021/9/24** | **-** | **-** | **S49** | **M** | **7** | **2021/12/3** | **+** | **+** | **S80** | **F** | **1** | **2022/1/14** | - | - |
| **S18** | **F** | **1** | **2021/9/26** | **+** | **+** | **S50** | **F** | **2** | **2021/12/6** | **+** | **+** | **S81** | **M** | **1** | **2022/2/20** | - | - |
| **S19** | **M** | **6** | **2021/9/27** | **-** | **-** | **S51** | **M** | **3** | **2021/12/4** | **-** | **-** | **S82** | **F** | **2** | **2022/2/26** | - | - |
| **S20** | **M** | **8** | **2021/10/1** | **-** | **-** | **S52** | **M** | **1** | **2021/12/4** | **+** | **+** | **S83** | **M** | **5** | **2022/3/6** | - | - |
| **S21** | **F** | **3** | **2021/9/26** | **-** | **-** | **S53** | **M** | **6** | **2021/12/6** | **-** | **-** | **S84** | **F** | **1** | **2022/3/16** | - | - |
| **S22** | **M** | **1** | **2021/10/3** | **-** | **-** | **S54** | **M** | **2** | **2021/12/6** | - | - | **S85** | **M** | **1** | **2022/3/21** | + | + |
| **S23** | **F** | **1** | **2021/9/24** | **-** | **-** | **S55** | **F** | **1** | **2021/12/6** | - | - | **S86** | **F** | **3** | **2022/4/10** | - | - |
| **S24** | **M** | **1** | **2021/10/4** | **-** | **-** | **S56** | **F** | **1** | **2021/12/8** | - | - | **S87** | **M** | **2** | **2022/4/16** | - | - |
| **S25** | **F** | **1** | **2021/9/23** | **-** | **-** | **S57** | **M** | **0.6** | **2021/12/12** | + | + | **S88** | **F** | **1.5** | **2022/4/18** | - | - |
| **S26** | **M** | **5** | **2021/9/23** | **+** | **+** | **S58** | **M** | **8** | **2021/12/13** | + | + | **S89** | **M** | **1** | **2022/5/8** | **-** | **-** |
| **S27** | **M** | **4** | **2021/9/27** | **-** | **-** | **S59** | **F** | **6** | **2021/12/16** | - | - | **S90** | **F** | **4** | **2022/4/25** | - | - |
| **S28** | **M** | **1** | **2021/9/24** | **-** | **-** | **S60** | **M** | **2** | **2021/12/14** | - | - | **S91** | **M** | **5** | **2022/4/28** | - | - |
| **S29** | **M** | **1** | **2021/9/28** | **-** | **-** | **S61** | **M** | **1** | **2021/12/14** | - | - | **S92** | **F** | **2** | **2022/5/24** | - | - |
| **S30** | **M** | **5** | **2021/9/29** | **-** | **-** | **S62** | **F** | **1** | **2021/12/16** | **-** | **-** | **S93** | **M** | **2** | **2022/5/28** | - | - |
| **S31** | **M** | **4** | **2021/9/26** | **-** | **-** | **S63** | **F** | **7** | **2021/12/18** | - | - | **S94** | **F** | **1** | **2022/5/26** | - | - |
| **S32** | **M** | **0.5** | **2021/10/1** | **-** | **-** |  |  |  |  |  |  |  |  |  |  |  |  |
